# ICTV Virus Taxonomy Profile: *Secoviridae*

**DOI:** 10.1099/jgv.0.000779

**Published:** 2017-04-28

**Authors:** Jeremy R. Thompson, Indranil Dasgupta, Marc Fuchs, Toru Iwanami, Alexander V. Karasev, Karel Petrzik, Hélène Sanfaçon, Ioannis Tzanetakis, René van der Vlugt, Thierry Wetzel, Nobuyuki Yoshikawa, ICTV Report Consortium

**Affiliations:** ^1^​ School of Integrative Plant Science, Cornell University, Ithaca, NY 14853, USA; ^2^​ Department of Plant Molecular Biology, University of Delhi South Campus, New Delhi 110021, India; ^3^​ School of Integrative Plant Science, Cornell University, New York State Agricultural Experiment Station, Geneva, NY 14456, USA; ^4^​ Apple Research Station, NARO Institute of Fruit Tree and Tee Science, Nabeyashiki 92-24, Shimokuriyagawa, Morioka, Iwate 020-0123, Japan; ^5^​ University of Idaho, Department of PSES, Moscow, ID 83844-2339, USA; ^6^​ Department of Plant Virology, Institute of Plant Molecular Biology, Biology Centre AS CR, Branisovska 31, 370 05 Ceske Budejovice, Czech Republic; ^7^​ Summerland Research and Development Centre, Agriculture and Agri-Food Canada, P.O. Box 5000, 4200 Highway 97, Summerland, B.C., Canada V0H 1Z0; ^8^​ Department of Plant Pathology, Division of Agriculture, University of Arkansas, Fayetteville, AR 72701, USA; ^9^​ Wageningen Research, Droevendaalsesteeg 1, Wageningen 6708 PB, The Netherlands; ^10^​ DLR Rheinpfalz - Institute of Plant Protection, Breitenweg 71, Neustadt an der Weinstrasse 67435, Germany; ^11^​ Plant Pathology Lab, Faculty of Agriculture, Iwate University, Ueda 3-18-8, Morioka 020-8550, Japan

**Keywords:** *Secoviridae*, ICTV report, taxonomy

## Abstract

Members of the family *Secoviridae* are non-enveloped viruses with mono- or bipartite (RNA-1 and RNA-2) linear positive-sense ssRNA genomes with the size of the RNAs combined ranging from 9 to 13.7 kb. They are related to picornaviruses and are classified in the order *Picornavirales*. The majority of known members infect dicotyledonous plants and many are important plant pathogens (e.g. grapevine fanleaf virus and rice tungro spherical virus). This is a summary of the current International Committee on Taxonomy of Viruses (ICTV) report on the taxonomy of the family *Secoviridae* available at www.ictv.global/report/secoviridae.

## Abbreviation

NTP, nucleotide triphosphate.

## Full-Text

**Table 1.** Characteristics of the family *Secoviridae*

Typical member:	cowpea mosaic virus (RNA-1: X00206; RNA-2: X00729), species *Cowpea mosaic virus*, genus *Comovirus*
Virion	Non-enveloped, 25–30 nm in diameter with icosahedral symmetry
Genome	9.0–13.7 kb of positive-sense, mono- or bipartite RNA
Replication	In association with intracellular membranes derived from the endoplasmic reticulum
Translation	Directly from genomic RNA as large polyproteins, which are cleaved by 3C-like proteinases
Host range	Plants (mainly dicots), transmitted mainly by insects or nematodes. Some seed transmission demonstrated
Taxonomy	In the order *Picornavirales*, family includes one subfamily with three genera, five additional genera and more than 70 species

## Virion

**Fig. 1.**
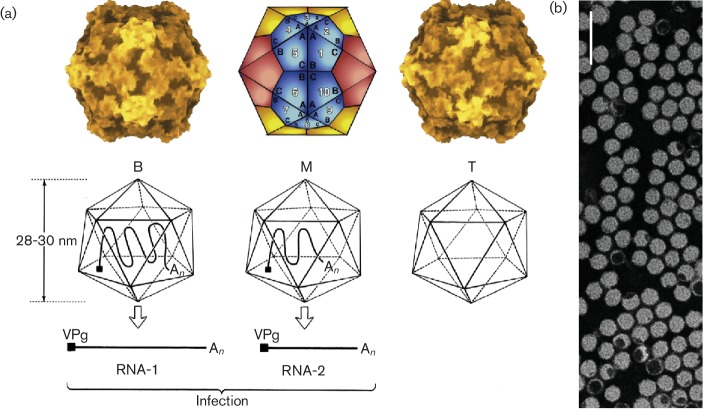
Virion structure and organization. (a) Top left: molecular rendering of the cowpea mosaic virus particle. Top centre: diagrammatic representation of a *T*=1 lattice. A, Small capsid protein; B, C-terminal domain of the large capsid protein; C, N-terminal domain of the large capsid protein. Top right: molecular rendering of the red clover mottle virus particle. Bottom: diagram of the three types of comovirus particles with the B-particle containing one molecule of RNA-1, the M-particle containing one molecule of RNA-2 and the T-particle being empty. (b) Negative contrast electron micrograph of particles of cowpea mosaic virus. The bar represents 100 nm.

Virions are non-enveloped, 25–30 nm in diameter and exhibit icosahedral symmetry (Table 1). Many virus preparations contain empty virus particles. In the case of viruses with a bipartite genome, the two RNAs are encapsidated in separate virions (Fig. 1) [1].

## Genome

**Fig. 2.**
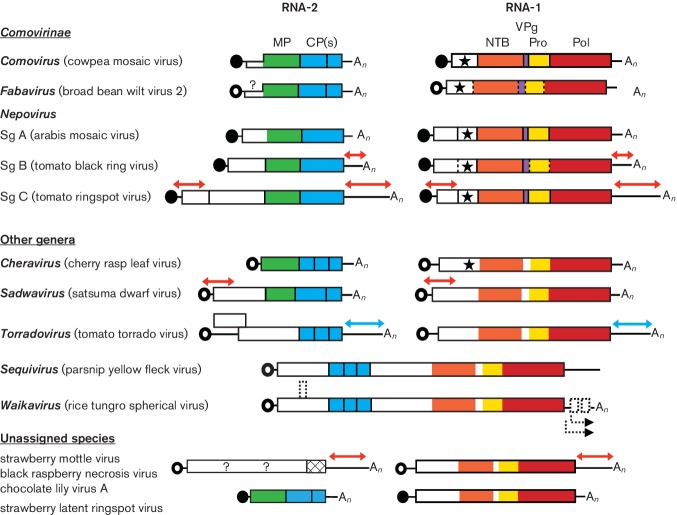
Genome organization of representative members of the family *Secoviridae*. Each RNA is shown with ORFs represented with boxes. Circles depict VPg molecules covalently attached at the 5′-end of the RNAs. Black circles represent VPg confirmed experimentally and open circles represent putative VPgs. Poly(A) tails are represented at the 3′-end of the RNAs when present (A_*n*_). Red and blue arrows above the sequences represent regions of extensive sequence identity between RNAs 1 and 2. In the latter, for torradoviruses, this identity is also characterized by conserved indels. Protein domains with conserved motifs for the putative NTP-binding protein (NTB, shown in orange), VPg (purple), proteinase (Pro, yellow), RNA-dependent RNA polymerase (Pol, red), movement protein (MP, green) and coat protein(s) (CP, blue) are shown. The star represents a conserved motif found in the protease cofactor (Co-Pro) protein of comoviruses and in the equivalent protein of other viruses. Proteinase cleavage sites identified experimentally or deduced by sequence comparisons are indicated by solid or dotted vertical lines, respectively. Possible ORFs in the genome of waikaviruses are shown with dotted rectangles and putative subgenomic RNAs are shown by dotted arrows below the waikavirus genome. Representatives of each nepovirus subgroup (Sg A, B, C) are also depicted.

The genome consists of one or two molecules of linear positive-sense ssRNA that are covalently linked to a small protein (viral protein genome-linked, VPg; 2–4 kDa) at their 5′ end and have a 3′-terminal poly(A) tract. Each RNA encodes, in the majority of the cases, a single polyprotein (Fig. 2).

## Replication

In the case of viruses with a bipartite genome, neither RNA species alone can infect plants systemically. Viral proteins are usually expressed as large polyproteins, which are cleaved by virus-encoded 3C-like proteinases. The replication block contains the domain characteristics of nucleoside triphosphate (NTP)-binding proteins (NTB or putative helicase), 3C-like proteinases (Pro) and RNA-dependent RNA polymerases (Pol) (Fig. 2). Replication occurs in association with intracellular membranes derived from the endoplasmic reticulum.

## Taxonomy

### Comovirus

Bipartite genome (subfamily *Comovirinae*). Comoviruses usually have narrow host ranges. Mosaic and mottle symptoms are characteristic. Transmission in nature is exclusively by beetles, especially members of the family Chrysomelidae. Beetles retain their ability to transmit virus for days or weeks [2].

### Fabavirus

Bipartite genome (subfamily *Comovirinae*). Fabaviruses have wide host ranges among dicotyledonous and some families of monocotyledonous plants. Symptoms are ringspots, mottling and wilting. In nature, they are transmitted by aphids in a non-persistent manner.

### Nepovirus

Bipartite genome (subfamily *Comovirinae*). The genus consists of >35 species that are widely distributed in temperate regions. Ringspot symptoms are characteristic. Many nepoviruses are transmitted non-persistently by longidorid nematodes. Seed and/or pollen transmission are also common. In herbaceous plants, the symptoms induced are often transient with a so-called ‘recovery’ phenomenon. The genus can be divided into subgroups (A, B, C) based on sequence and genome organization [3].

### Cheravirus

Bipartite genome. Symptoms are usually mild or absent. Cherry rasp leaf virus is transmitted by nematodes in the field [4].

### Sadwavirus

Bipartite genome, only one species, *Satsuma dwarf viru*s, members of which have a wide host range. The natural mode of transmission is unknown [5].

### Torradovirus

Bipartite genome. RNA-2 contains an ORF upstream and partially overlapping the large ORF. Some torradoviruses are known to be transmitted by whiteflies in a semi-persistent manner. Aphid transmission has been demonstrated for carrot torrado virus 1 [6].

### Sequivirus

Monopartite genome. The natural host range of sequiviruses includes plants in several families. Transmission is by aphids in a semi-persistent manner. However, it is dependent on the presence of a helper virus in the genus *Waikavirus*.

### Waikavirus

Monopartite genome. The natural host range of waikaviruses is usually restricted to plants within a few families. Field transmission is semi-persistent by aphids or leafhoppers. Some waikaviruses are helper viruses for the insect transmission of other viruses; for example, rice tungro spherical virus is the helper virus for rice tungro bacilliform virus (family *Caulimoviridae*).

## Resources

Full ICTV Online (10th) Report: www.ictv.global/report/secoviridae.
